# Novel designed balloon specialized for EUS-guided gastroenterostomy: A pilot study

**DOI:** 10.1097/eus.0000000000000129

**Published:** 2025-11-03

**Authors:** Shimin Wang, Pingping Zhang, Hangyu Chen, Ping Li, Bo Li, Xiangyu Kong, Yu Zhang, Ting Yang, Jing Sun, Zhendong Jin, Kaixuan Wang

**Affiliations:** 1 Department of Gastroenterology, Ruijin Hospital, Shanghai Jiao Tong University School of Medicine, Shanghai, China; 2 Department of Gastroenterology, Changhai Hospital, The First Affiliated Hospital of Naval Medical University, Shanghai, China; 3 Department of Anesthesia, Changhai Hospital, The First Affiliated Hospital of Naval Medical University, Shanghai, China.

**Keywords:** EUS, Gastroenterostomy, Balloon-assisted device, Gastric outlet obstruction, Gastric cancer

## Abstract

**Background and objectives:**

We recently developed a balloon-assisted device for EUS-guided gastroenterostomy (EUS-GE) to enhance the safety and convenience of the procedure. This pilot study was conducted to evaluate the safety and feasibility of this device.

**Methods:**

A retrospective analysis of data of patients who underwent EUS-GE using this balloon-assisted device at our institution from March 2024 to July 2024 was conducted. The primary end point was the procedure time, and the secondary end points were the volume of water injection, technical success rate, clinical success rate, and adverse events (AEs).

**Results:**

A total of 20 patients (male: 55%; female: 45%) were enrolled, with a mean age of 67.7 ± 9.9 years. The mean procedure time was 29.3 ± 9.4 minutes, and the mean intraoperative water infusion in the jejunum was 92.5 (80–117.5) mL. The technical success rate was 100% (20/20). The clinical success rate was 95% (19/20). One patient (5%) experienced mild abdominal pain after the procedure. No other AEs, such as bleeding, perforation, stent occlusion, or migration, were observed during follow-up. The median follow-up duration was 132 (74–170) days.

**Conclusion:**

The balloon-assisted device facilitates the application of EUS-GE, with short procedure time, less intraoperative water injection, high technical success rate, and low incidence of AEs.

## Background

EUS-guided gastroenterostomy (EUS-GE) is an emerging technique for the treatment of malignant gastric outlet obstruction (GOO). Compared with surgery, EUS-GE is less invasive and has fewer adverse events (AEs).^[1–3]^ In addition, it has a lower need for reintervention and a lower rate of stent dysfunction compared with traditional enteral stent (ES) implantation.^[4–6]^ Consequently, EUS-GE has been included in the recent European Society of Gastrointestinal Endoscopy guidelines as an alternative to ES placement or surgical gastrojejunostomy (SGJ).^[7]^ Currently, EUS-GE is performed through 3 main methods: direct approach, balloon-assisted approach, and EUS-guided double-balloon–occluded gastrojejunostomy bypass (EPASS). The direct approach uses a large volume of fluid, which may not be suitable for patients with cardiovascular and renal diseases. The EPASS is expensive and therefore not yet widely used. The balloon-assisted approach is less stable because the balloon needs to be punctured with a needle, and there is a risk of pushing the target intestine away during guide wire placement. In addition, all these 3 methods require advanced endoscopic techniques and hence not conducive to clinical application. To overcome these challenges, our team has designed a balloon-assisted device specifically for EUS-GE to shorten the procedure time, reduce the volume of intraoperative water injection, and improve the procedural safety.^[8]^ The present study aims to verify the feasibility and safety of our balloon-assisted device in clinical practice.

## Methods

### Study design and population

A single-center retrospective analysis of the clinical data of patients with malignant GOO who underwent EUS-GE using the novel balloon-assisted device was conducted between March 2024 and July 2024. Inclusion criteria were as follows: (1) age of 18–80 years old; (2) diagnosed as malignant GOO; (3) patients with malignant GOO who are not suitable or unable to tolerate surgery; (4) there were no absolute contraindications to EUS-GE; and (5) informed consent was obtained. Exclusion criteria were as follows: (1) pregnant and lactating women; (2) no informed consent was obtained; (3) cognitive impairment, aphasia, mental disorders or diseases that may affect patient cooperation; (4) patients with endoscopy contraindications, anesthesia contraindications, gastrointestinal perforation, and abdominal infection; (5) not considered suitable for the participant by the investigator.

The primary endpoint was the procedure time. The secondary endpoints were the volume of water injected during the procedure, technical success rate, clinical success rate, and AE rate. Procedure time was defined as the time from the endoscopic detection of GOO to the end of the EUS-GE procedure, including the identification of the puncture site, the performance of the puncture, and the successful placement of the lumen-apposing metal stent (LAMS).^[9–11]^ Technical success was defined as the adequate deployment of the LAMS confirmed by a combination of endoscopy, endosonography, and fluoroscopy.^[12]^ Clinical success was defined as a Gastric Outlet Obstruction Scoring System (GOOSS) score of ≥2 within 1 week of the procedure.^[5]^ AEs were defined according to the American Society for Gastrointestinal Endoscopy lexicon.^[13]^

### Novel device

**Figure 1:**
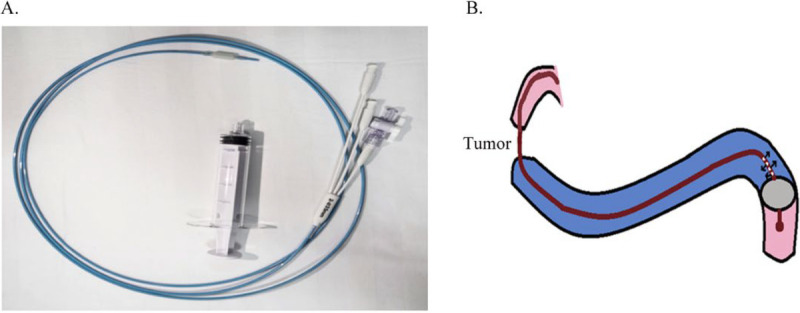
A, Novel balloon-assisted device. B, Schematic diagram showing the placement of the novel balloon-assisted device in the first part of the jejunum followed by distension of the proximal bowel loop with water.

The technical details of the EUS-GE have been previously described in detail by our research team.^[8]^ This procedure was performed using a curved linear array echo endoscopy (Fujifilm SU-9000, Tokyo, Japan). This novel balloon (Micro-Tech, Nanjing, China) has been approved in China. The outer tube diameter of the device is 7F, the effective length is 2.3 m, and the front section has a large balloon, which is about 4 cm in diameter after inflation, which can effectively fix the target intestine. In addition, the balloon has a water injection hole at the rear end, which is convenient for injecting normal saline to fill the target intestine (Figure 1A).

### Procedure and postoperative treatment

All patients received prophylactic dose of second-generation cephalosporin preprocedure. General anesthesia with tracheal intubation was administered, and the patient was placed in prone position. Initially, a contrast medium was injected under direct gastroscopic visualization to identify the site and length of the stenosis. A guide wire was then placed into the proximal jejunum, along the contrast catheter. The novel balloon device was inserted over the guide wire, and about 40 mL of air was injected into the balloon to ensure proper inflation under fluoroscopic guidance. The segment of the jejunum proximal to the balloon was distended with 1:1 dilution of contrast medium and methylene blue (Figure 1B). A linear-array echoendoscope was inserted into the midbody of the stomach along the greater curvature to visualize the distended segment of the jejunum. Under EUS guidance, a HOT-AXIOS 1.5 × 1.0-cm LAMS metal stent was deployed. During the stent release, the methylene blue was observed overflowing from the jejunum into the stomach. Fluoroscopy was used to confirm good stent expansion, with the contrast medium passing through the stent into the distal jejunum. The balloon and echoendoscope were then removed, completing the procedure. All patients were kept nil per oral for 24 hours postoperatively, after which the diet was gradually advanced as tolerated.

### Follow-up

The patients were followed up until December 1, 2024, or death. The following parameters were monitored: (1) GOOSS score; (2) complications related to the procedure, including abdominal pain, infection, stent migration, stent occlusion, and mortality; and (3) overall postoperative quality of life (QoL) of the patients.

### Statistical analysis

Results were reported as mean (SD) and median (interquartile range [IQR]) for quantitative variables and percentages for categorical variables. If the patient was lost to follow-up or alive at the time of the study, the date of the last follow-up was used to estimate survival as censored data with the Kaplan-Meier method.

## Results

### Baseline characteristics

A total of 20 patients (male: 55%; female: 45%) were enrolled. The median age of the patients enrolled was 67.7 ± 9.9 years. The common etiologies of malignant GOO were pancreatic malignancy (75%), gastric antrum malignancy (10%), and duodenal malignancy (15%). Preoperatively, 4 patients had a small amount of ascites, 14 patients had a GOOSS score of 0, and 6 patients had a GOOSS score of 1. A small amount of ascites was noted in 4 patients. Lymph node metastasis was noted in 16 patients. Six patients had received chemotherapy before the endoscopic intervention, of which 5 patients were on the AG regimen and 1 patient was on the mFOLFIRINOX regimen (Table 1).

**Table 1:** Baseline characteristics.

Age, mean ± SD, yr	67.7 ± 9.9
Female sex, *n* (%)	9 (45)
Malignant etiology, *n* (%)	
Pancreatic cancer	15 (75)
Gastric cancer	2 (10)
Duodenal cancer	3 (15)
Ascites, *n* (%)	4 (20)
Preoperative GOOSS score, *n* (%)	
0	14 (70)
1	6 (30)
2	0 (0)
3	0 (0)
Lymphatic metastasis, *n*(%)	16 (80)
Chemotherapy, *n* (%)	6 (30)
AG	5 (25)
mFOLFIRINOX	1 (5)

### Operation outcomes

The average procedure time was 29.3 ± 9.4 minutes. The technical success rate was 100% (20/20). In addition, 92.5 (80–117.5) mL of water was intraoperatively injected. The clinical success rate was 95% (19/20), with 1 patient showing no improvement in the GOOSS score postoperatively. The mean length of stay was 8 (5–13) days. One patient (5%) experienced mild postoperative abdominal pain; the patient was resolved with conservative management and discharged without any bleeding, perforation, stent occlusion, stent migration, or other AEs. Four patients died due to progression of the primary malignancy (Table 2).

**Table 2:** Outcomes.

Procedure time, (Mean ± SD, minutes)	29.3 ± 9.4
Infusion volume, (mL)	92.5 (80–117.5)
Technical success, *n* (%)	20 (100)
Clinical success, *n* (%)	19 (95)
Postoperative GOOSS score, *n* (%)	
0	0 (0)
1	1 (5)
2	13 (65)
3	6 (30)
Length of stay, d	8 (5–13)
Adverse events, *n* (%)	
Abdominal pain	1 (5)
Bleeding	0 (0)
Perforation	0 (0)
Stent occlusion	0 (0)
Stent migration	0 (0)
Others	0 (0)
Mortality, *n* (%)	4 (20)

### Follow-up

**Figure 2:**
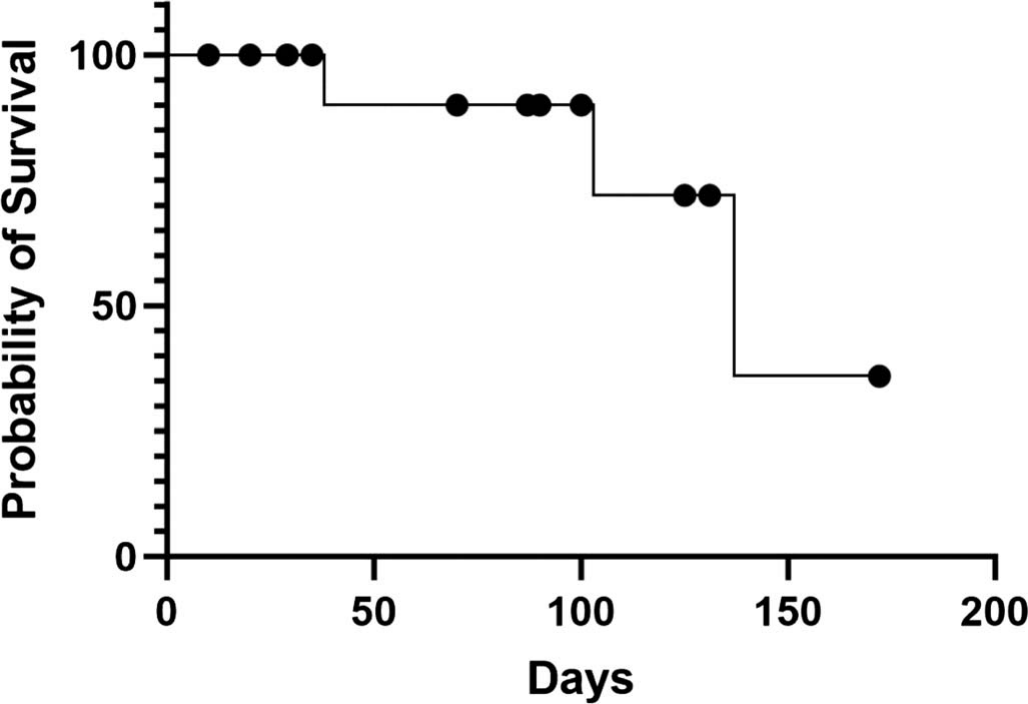
Patient survival curve.

The median follow-up time for the patients was 132 (74–170) days. Four deaths were reported by the follow-up date (mortality rate of 20%), all due to the progression of the primary tumor. The median survival time was 137 (87–187) days. The survival curve is detailed in Figure 2. During the follow-up period, the QoL was regularly assessed. The median QoL scores were 39 (34–45) before treatment and 53 (44–56) at 4 weeks after the treatment (Table 3).

**Table 3:** Quality-of-life self-assessment using the QoL score.

	QoL score
Before the procedure	39 (34–45)
After week 4	53 (44–56)

## Discussion

EUS-GE is an evolving interventional EUS technique for symptomatic GOO. Currently, many studies have compared EUS-GE with endoscopic stent placement. They found no significant differences in technical success rates (EUS-GE: 86.7% *vs.* ES: 94.2%, *P* = 0.2), clinical success rates (83.3% *vs.* 67.3%, *P* = 0.12), AE rates (16.7% *vs.* 11.5%, *P* = 0.5), and serious AE rates (10% *vs.* 9.6%, *P* = 0.95) between the 2 procedures.^[14]^ Additionally, when compared with SGJ, the technical (97.9% *vs.* 100%) and clinical (94.1% *vs.* 94.3%) success rates of EUS-GE were similar. The AE rate for EUS-GE was lower (13.4% *vs.* 33.3%, *P* < 0.001), but the reintervention rate was higher (15.5% *vs.* 1.63%, *P* < 0.001). EUS-GE resulted in a significant reduction in the time to resume oral intake compared with SGJ (1.40 *vs.* 4.06 days, *P* < 0.001). It is also associated with shorter hospital stay (5.31 *vs.* 8.54 days, *P* < 0.001).^[3]^

In 2012, Binmoeller and Shah first described EUS-GE for the treatment of GOO.^[15]^ The application of the Hot AXIOS stent deployment system, which incorporates electrocautery, has made the EUS-GE technique safer and more efficient.^[15]^ After a decade of development, EUS-GE has now been included in the recent European Society of Gastrointestinal Endoscopy guidelines. However, identifying the target intestinal segment is crucial for the successful deployment of stents. Despite the development and refinement of various assistive technologies for EUS-GE, an optimal technique has not been developed.

At present, the direct EUS-GE technique is widely used for EUS-GE. A nasobiliary or anasojejunal nutrition tube is inserted along the guide wire across the GOO, and water is continuously injected into the duodenum and proximal jejunum. A linear echoendoscope is used to visualize the saline-filled duodenum or jejunum. A LAMS with an electrocautery-enhanced delivery system, such as the AXIOS stent, is used to establish a tract and deploy the stent.^[7]^ The technical success rate of the direct method is 94.2% to 97.1%,^[14,16]^ with an operation time of 35.7 ± 32.1 minutes.^[14]^ When a large amount of water is injected into the small intestine, it induces peristalsis, which can hamper the stability of the target jejunal loop. Moreover, the jejunal loop often collapses due to peristalsis despite antispasmodic medications. In addition, the injection of a large amount of fluid into the small intestine for a long time can dilate the distal small bowel loops and transverse colon, resulting in incorrect puncture direction, which may lead to poor puncture path or penetration into the colon,^[17]^ resulting in incorrectly deployed stents with type III or type IV stent complications.^[18]^ Also, the large and rapid injection of 500 mL or more water during endoscopic intervention^[14]^ may lead to fluid overload in patients with cardiovascular and renal diseases.

In the balloon-assisted technique, under the guidance of EUS, the balloon is punctured through the gastric wall with a fine-needle aspiration needle, and the guide wire was placed in the distal jejunum along the puncture needle core. Next, a fistula tract is created along the guide wire, and a stent is deployed.^[7]^ The technical success rate of the balloon-assisted is 88.9% to 90.9%,^[14,19]^ with a procedure time of 89.9 ± 33.3 minutes.^[14]^ Compared with other methods, the procedure time of the balloon-assisted approach is longer because the fistula tract is established solely with the support of a guide wire and does not have a high stability due to which the target bowel tube may get pushed away during stent placement.^[17]^

The EPASS was first reported in 2013 by Itoi et al., who successfully applied it in pigs.^[20]^ The procedure involves advancing the endoscope to the stenosis point, inserting the guide wire through the Treitz ligament and advancing the double balloon along the guide wire with fluoroscopic guidance. The best location for the puncture is selected, and an electrocautery-enhanced LAMS is implanted.^[12,21]^ The technical success rate of the EPASS method is 91% to 94.6%, with a procedure time of 27.3 to 64.8 minutes.^[12,21]^ The EPASS procedure is relatively short in duration, and the puncture target is stable, facilitating jejunal puncture. However, the distance between the 2 balloons is 9 cm, and the position of the target intestine must be carefully assessed during the procedure. Additionally, inflating both balloons sequentially is time-consuming and requires a high level of skill.^[22]^ Currently, EPASS devices are available only in some Asian countries and are expensive, which limits their clinical application.

**Figure 3:**
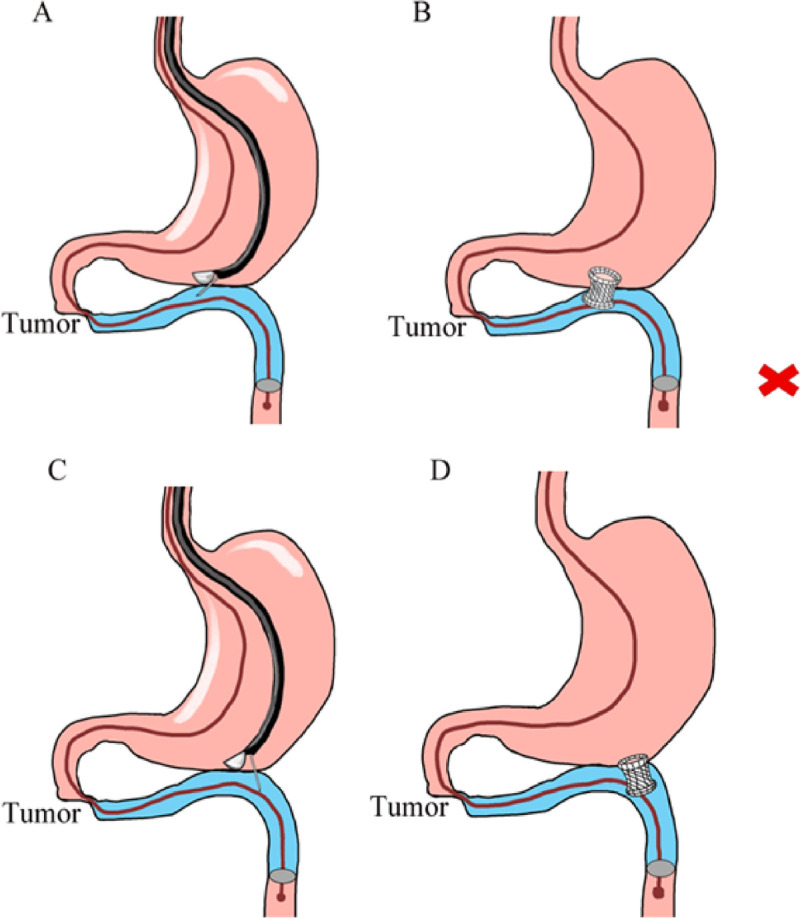
Balloon-assisted EUS-GE. A, After inflation of the balloon, the proximal jejunum is punctured at a suitable point. B, Avoid release of the flange toward the proximal jejunum. C, The puncture site must be toward the distal jejunum. D, The stent is deployed in the distal jejunum.

Our novel balloon-assisted device is easy to operate and simplifies the procedure. The balloon is placed into the distal jejunum, and the intestinal cavity is blocked by injecting gas in the balloon. After the balloon is placed, the intestinal tube can be efficiently filled with only a small amount of water, which eases the identification of the target intestinal segment. The clinical success rate of this technique was 95% in our study, which was consistent with the clinical success rate of other methods reported by Tsuchiya et al. and Vanella et al. (89%–97.1%).^[12,14,16,21]^ In addition, with our balloon, the direction of endoscopic puncture can be adjusted during the procedure. We chose our puncture site in the distal jejunum, which is conducive to the smooth passage of food through the gastric stent into the intestine (Figure 3). Therefore, our balloon-assisted device improved GOOSS score of patients, and only one patient in our report did not have improvement in GOOSS score. The remaining 95% of patients had significant improvement in GOOSS.

The average procedure time for EUS-GE using our novel balloon-assisted device was 29.3 ± 9.4 minutes, with a technical success rate of 100%, consistent with other literature reports (90.9%–97.1%).^[12,14,16,21]^ Notably, the mean infusion volume was 92.5 (80–117.5) mL, significantly reducing the risk for patients with cardiovascular disorders.

The novel balloon-assisted device evidently enhances the confidence of endoscopists. A survey conducted among clinicians performing EUS-GE in our department indicated that this device was easy to use and comfortable, with operators expressing a willingness to continue using this technique and also to teach its usage to others. Although previous studies have reported a faster recovery of oral intake with a 20-mm stent,^[23]^ this stent is not available in China. Therefore, we utilized a 15-mm LAMS. An analysis of postoperative GOOSS scores revealed that the 15-mm stent significantly improves patients’ QoL, with median QoL scores of 39 (34–45) before treatment and 53 (44–56) at 4 weeks after treatment. In a prospective study, a clinically significant increase of 21.6 points (95% confidence interval, 11.5–31.7) in the global health status scale was documented, with significant improvements in nausea and vomiting, pain, constipation, and appetite loss.^[24]^ Consistent with our report, both showed improved QoL after EUS-GE. Previous studies reported a median postoperative survival time of 51 days (range, 8–417 days) for GOO treated with EUS-GE.^[25]^ In the current study, the median postoperative survival time was 137 days (87–187 days).

Postoperative mild abdominal pain, bleeding, stent occlusion, and stent loss are common AEs after EUS-GE, with an incidence of 12.9% to 16.2%.^[16,21]^ In this study, only one patient had mild abdominal pain, which improved after conservative treatment, and no stent-related AEs were found. In our study, 12 patients received chemotherapy after EUS-GE, and previous studies have reported that EUS-GE allows for earlier resumption of chemotherapy than SGJ.^[3]^

Misdeployed stents are a factor hindering the promotion of EUS-GE treatment for GOO, which occurs in 9.85% of patients after the procedure. Type I was the most common type of misdeployed stent (63.1%), followed by type II (30.4%) in 14 cases, type IV (4.3%) in 2 cases, and type III (2.2%) in 1 case.^[18]^ It is worth mentioning that although no types II and III complications occurred in the study patients. If types II and III complications occur during the procedure, then we can withdraw the balloon to the proximal end of the perforation to seal the intestinal tube. At the same time, negative pressure attracts the proximal digestive tract fluid and keeps the perforation site dry, which will be more conducive for the closure of the perforation (Supplementary Figure 1, http://links.lww.com/ENUS/A380).

There are some limitations to this study. First, it is a single-center retrospective study. Second, there was no comparative group with other methods of the outcomes, such as the direct approach or EPASS. Third, the sample size was small. We have applied for prospective randomized controlled trials to compare the safety and efficacy of this novel balloon-assisted device and the direct approach in the treatment of malignant GOO.

In conclusion, our novel device is safe and effective for EUS-GE in patients with malignant GOO. It also reduces the procedure time, increases the technical success rate, and minimizes the intraoperative infusion volume required for EUS-GE.

## Source of Funding

This study was supported by Sanming Project of Medicine in Shenzhen (no. 1) and The Incubation Program for Clinical New Technologies in 2024 at Changhai Hospital Affiliated with the Naval Medical University (reference no. 2024XC103).

## Ethical Approval

This study was approved by Shanghai Changhai hospital hospital Medical Ethics Committee (approval CHEC2025-284).

## Informed Consent

Informed consents were obtained.

## Conflict of Interest

Zhendong Jin is an Associate Editor of the journal. This article was subject to the journal’s standard procedures, with peer review handled independently of the editor and his research group. The authors declare that they have no financial conflict of interest with regard to the content of this report.

## Author Contributions

K. Wang takes responsibility for all aspects of the work, ensuring that any questions related to the accuracy or integrity of any part of the work are appropriately investigated and resolved. S. Wang, P. Zhang, and H. Chen contributed to the acquisition and analysis of data, and drafted the manuscript. S. Wang performed the visualization of the data. J. Sun and S. Wang were involved in the review and editing of the manuscript. Z. Jin and K. Wang provided final approval of the version to be published. The others critically revised the manuscript for important intellectual content. All authors have read and approved the final manuscript.

## Data Availability Statement

The datasets used and/or analyzed during the current study are available from the corresponding author on reasonable request.

## References

[1] Abbas A Dolan RD Bazarbashi AN Thompson CC. Endoscopic ultrasound–guided gastroenterostomy *versus* surgical gastrojejunostomy for the palliation of gastric outlet obstruction in patients with peritoneal carcinomatosis. *Endoscopy* 2022;54(7):671–679. 35120397 10.1055/a-1708-0037

[2] Kumar A Chandan S Mohan BP, . EUS-guided gastroenterostomy *versus* surgical gastroenterostomy for the management of gastric outlet obstruction: A systematic review and meta-analysis. *Endosc Int Open* 2022;10(4):E448–e58. 35433208 10.1055/a-1765-4035 PMC9010090

[3] Canakis A Bomman S Lee DU, . Benefits of EUS-guided gastroenterostomy over surgical gastrojejunostomy in the palliation of malignant gastric outlet obstruction: A large multicenter experience. *Gastrointest Endosc* 2023;98(3):348–59.e30. 37004816 10.1016/j.gie.2023.03.022

[4] Asghar M Forcione D Puli SR. Endoscopic ultrasound–guided gastroenterostomy *versus* enteral stenting for gastric outlet obstruction: A systematic review and meta-analysis. *Therap Adv Gastroenterol* 2024;17:17562848241248219. 10.1177/17562848241248219 PMC11159541 38855340

[5] Conti Bellocchi MC Gasparini E Stigliano S, . Endoscopic ultrasound–guided gastroenterostomy *versus* enteral stenting for malignant gastric outlet obstruction: A retrospective propensity score–matched study. *Cancers (Basel)* 2024;16(4):724. 38398115 10.3390/cancers16040724 PMC10887005

[6] Monino L Perez-Cuadrado-Robles E Gonzalez JM, . Endoscopic ultrasound–guided gastroenterostomy with lumen-apposing metal stents: A retrospective multicentric comparison of wireless and over-the-wire techniques. *Endoscopy* 2023;55(11):991–999. 37380033 10.1055/a-2119-7529

[7] van der Merwe SW van Wanrooij RLJ Bronswijk M, . Therapeutic endoscopic ultrasound: European Society of Gastrointestinal Endoscopy (ESGE) guideline. *Endoscopy* 2022;54(2):185–205. 34937098 10.1055/a-1717-1391

[8] Wang KX Zhang PP Yang T Zhang Y. Endoscopic ultrasonography–guided single balloon-occluded gastrojejunostomy bypass: A case report. *Endoscopy* 2024;56(S 01):E860–e1. 39401755 10.1055/a-2414-7602 PMC11473184

[9] Bronswijk M Vanella G van Malenstein H, . Laparoscopic *versus* EUS-guided gastroenterostomy for gastric outlet obstruction: An international multicenter propensity score–matched comparison (with video). *Gastrointest Endosc* 2021;94(3):526–36.e2. 33852900 10.1016/j.gie.2021.04.006

[10] Jovani M Ichkhanian Y Parsa N, . Assessment of the learning curve for EUS-guided gastroenterostomy for a single operator. *Gastrointest Endosc* 2021;93(5):1088–1093. 32991868 10.1016/j.gie.2020.09.041

[11] Xu G Shen Y Lv Y, . Safety and efficacy of endoscopic ultrasound–guided gastroenterostomy using double balloon occlusion methods: A clinical retrospective study in 36 patients with malignant gastric outlet obstruction. *Endosc Int Open* 2020;8(11):E1690–e7. 33140026 10.1055/a-1221-9656 PMC7581485

[12] Marino A Bessissow A Miller C, . Modified endoscopic ultrasound–guided double-balloon-occluded gastroenterostomy bypass (M-EPASS): A pilot study. *Endoscopy* 2022;54(2):170–172. 33592629 10.1055/a-1392-4546

[13] Cotton PB Eisen GM Aabakken L, . A lexicon for endoscopic adverse events: Report of an ASGE workshop. *Gastrointest Endosc* 2010;71(3):446–454. 20189503 10.1016/j.gie.2009.10.027

[14] Chen YI Kunda R Storm AC, . EUS-guided gastroenterostomy: A multicenter study comparing the direct and balloon-assisted techniques. *Gastrointest Endosc* 2018;87(5):1215–1221. 28750837 10.1016/j.gie.2017.07.030

[15] Binmoeller KF Shah JN. Endoscopic ultrasound–guided gastroenterostomy using novel tools designed for transluminal therapy: A porcine study. *Endoscopy* 2012;44(5):499–503. 22531985 10.1055/s-0032-1309382

[16] Vanella G Dell'Anna G Capurso G, . EUS-guided gastroenterostomy for management of malignant gastric outlet obstruction: A prospective cohort study with matched comparison with enteral stenting. *Gastrointest Endosc* 2023;98(3):337–47.e5. 37094692 10.1016/j.gie.2023.04.2072

[17] Carbajo AY Kahaleh M Tyberg A. Clinical review of EUS-guided gastroenterostomy (EUS-GE). *J Clin Gastroenterol* 2020;54(1):1–7. 10.1097/MCG.0000000000001262 31567785

[18] Ghandour B Bejjani M Irani SS, . Classification, outcomes, and management of misdeployed stents during EUS-guided gastroenterostomy. *Gastrointest Endosc* 2022;95(1):80–89. 34352256 10.1016/j.gie.2021.07.023

[19] Khashab MA Kumbhari V Grimm IS, . EUS-guided gastroenterostomy: The first U.S. clinical experience (with video). *Gastrointest Endosc* 2015;82(5):932–938. 26215646 10.1016/j.gie.2015.06.017

[20] Itoi T Itokawa F Uraoka T, . Novel EUS-guided gastrojejunostomy technique using a new double-balloon enteric tube and lumen-apposing metal stent (with videos). *Gastrointest Endosc* 2013;78(6):934–939. 24237949 10.1016/j.gie.2013.09.025

[21] Tsuchiya T Itoi T Ishii K, . Long-term outcomes of endoscopic ultrasonography–guided balloon-occluded gastrojejunostomy bypass for malignant gastric outlet obstruction (with video). *Gastrointest Endosc* 2025;101(1):195–199. 39053650 10.1016/j.gie.2024.07.006

[22] Bronswijk M Pérez-Cuadrado-Robles E Van der Merwe S. Endoscopic ultrasound–guided gastrointestinal anastomosis: Current status and future perspectives. *Dig Endosc* 2023;35(2):255–263. 35726383 10.1111/den.14381

[23] Bejjani M Ghandour B Subtil JC, . Clinical and technical outcomes of patients undergoing endoscopic ultrasound–guided gastroenterostomy using 20-mm *vs.* 15-mm lumen-apposing metal stents. *Endoscopy* 2022;54(7):680–687. 34569611 10.1055/a-1654-6914

[24] Garcia-Alonso FJ Chavarria C Subtil JC, . Prospective multicenter assessment of the impact of EUS-guided gastroenterostomy on patient quality of life in unresectable malignant gastric outlet obstruction. *Gastrointest Endosc* 2023;98(1):28–35. 36801458 10.1016/j.gie.2023.02.015

[25] Basha J Lakhtakia S Yarlagadda R, . Gastric outlet obstruction with ascites: EUS-guided gastro-enterostomy is feasible. *Endosc Int Open*. 2021;9(12):E1918–e23. 34917463 10.1055/a-1642-7892 PMC8670992

